# Excellent Diagnostic Characteristics for Ultrafast Gene Profiling of *DEFA1-IL1B-LTF* in Detection of Prosthetic Joint Infections

**DOI:** 10.1128/JCM.00558-17

**Published:** 2017-08-23

**Authors:** Regina Fillerova, Jiri Gallo, Martin Radvansky, Veronika Kraiczova, Milos Kudelka, Eva Kriegova

**Affiliations:** a Department of Immunology, Faculty of Medicine and Dentistry, Palacky University & University Hospital, Olomouc, Czech Republic; b Deptartment of Orthopaedics, Faculty of Medicine and Dentistry, Palacky University, Olomouc, Czech Republic; c Department of Computer Science, Faculty of Electrical Engineering and Computer Science, VSB-Technical University of Ostrava, Ostrava, Czech Republic; Mayo Clinic

**Keywords:** prosthetic joint infection, gene expression, pseudosynovial tissues, diagnostics, intraoperative test

## Abstract

The timely and exact diagnosis of prosthetic joint infection (PJI) is crucial for surgical decision-making. Intraoperatively, delivery of the result within an hour is required. Alpha-defensin lateral immunoassay of joint fluid (JF) is precise for the intraoperative exclusion of PJI; however, for patients with a limited amount of JF and/or in cases where the JF is bloody, this test is unhelpful. Important information is hidden in periprosthetic tissues that may much better reflect the current status of implant pathology. We therefore investigated the utility of the gene expression patterns of 12 candidate genes (*TLR1*, -*2*, -*4*, -*6*, and *10*, *DEFA1*, *LTF*, *IL1B*, *BPI*, *CRP*, *IFNG*, and *DEFB4A*) previously associated with infection for detection of PJI in periprosthetic tissues of patients with total joint arthroplasty (TJA) (*n* = 76) reoperated for PJI (*n* = 38) or aseptic failure (*n* = 38), using the ultrafast quantitative reverse transcription-PCR (RT-PCR) Xxpress system (BJS Biotechnologies Ltd.). Advanced data-mining algorithms were applied for data analysis. For PJI, we detected elevated mRNA expression levels of *DEFA1* (*P* < 0.0001), *IL1B* (*P* < 0.0001), *LTF* (*P* < 0.0001), *TLR1* (*P* = 0.02), and *BPI* (*P* = 0.01) in comparison to those in tissues from aseptic cases. A feature selection algorithm revealed that the *DEFA1-IL1B-LTF* pattern was the most appropriate for detection/exclusion of PJI, achieving 94.5% sensitivity and 95.7% specificity, with likelihood ratios (LRs) for positive and negative results of 16.3 and 0.06, respectively. Taken together, the results show that *DEFA1-IL1B-LTF* gene expression detection by use of ultrafast qRT-PCR linked to an electronic calculator allows detection of patients with a high probability of PJI within 45 min after sampling. Further testing on a larger cohort of patients is needed.

## INTRODUCTION

Prosthetic joint infection (PJI) is one of the most devastating and costly complications following total joint arthroplasty (TJA), occurring in 1 to 2% of cases after primary TJA and up to 7% of cases following revision TJA (1).

The majority of PJIs are easily diagnosed using the traditional diagnostic armamentarium (2). However, there are cases where the diagnosis of PJI lacks the required accuracy and/or the results of blood and joint fluid tests are unavailable or conflicting. For some patients, joint fluid for preoperative examinations is not even available. On the other hand, patients with systemic inflammatory diseases may have elevated inflammatory markers regardless of the presence or absence of infection (3). Finally, in some cases, the probability of PJI can increase just after pseudocapsule incision or removal of the implant on the basis of the appearance of periprosthetic tissues and/or joint fluid, regardless of the results of preoperative tests. Taken together, these instances indicate that there is a strong requirement for a diagnostic tool that is exact and available in a timely manner for the intraoperative exclusion of PJI.

At present, there is evidence of the usefulness of the leukocyte-esterase test and, especially, the alpha-defensin lateral immunoassay for the intraoperative exclusion of PJI, both of which deliver results within 10 min (4). However, these tests have well-known limitations in terms of the required working conditions (e.g., the minimum amount of joint fluid needed and a problem with blood interference) and the generation of false-positive and/or false-negative results (5–8). Periprosthetic tissues should also be considered an important source of diagnostic information. The cells within the tissue may directly reflect the current status of the infection, e.g., by the production of host defense antimicrobial peptides and proteins and activation of an innate immune response. Importantly, pseudosynovial and periprosthetic tissues are easily available at the beginning of surgery for further analysis; they are now used mainly for routine culture examination of bacteria (9). Some studies have examined the clinical utility of frozen sections, with clear limitations (10). Recently, it was reported that gene profiling of periprosthetic tissues may allow the proposal of novel PJI biomarkers, as shown for *TLR1* (11).

We therefore investigated the expression patterns of 12 candidate genes for detection of PJI in periprosthetic tissues of patients with TJA. The candidate genes were selected from promising biomarkers of PJI reported previously for (i) synovial fluid, namely, *DEFA1* (coding for alpha-defensin), *LTF* (lactotransferrin), *CRP* (C-reactive protein), *BPI* (bactericidal/permeability-increasing protein), *IL1B* (interleukin 1 beta), *DEFB4A* (beta-defensin 4), and *IFNG* (gamma interferon) (4, 12), and (ii) periprosthetic tissue and serum, namely, *TLR1* (Toll-like receptor 1), *TLR2*, *TLR4*, *TLR6*, and *TLR10* (11, 13, 14). Moreover, we studied whether ultrafast quantitative one-step reverse transcription-PCR (qRT-PCR) may deliver results within a reasonable time (up to 45 min) after sampling, thus helping surgeons in decision-making during the operation.

## RESULTS

### Gene expression profiling.

In order to determine PJI-associated gene expression patterns in periprosthetic tissues, we compared gene expression levels of 12 candidate molecules (*TLR1*, -*2*, -*4*, -*6*, and -*10*, *DEFA1*, *LTF*, *IL1B*, *BPI*, *CRP*, *IFNG*, and *DEFB4A*) previously reported as potential biomarkers for detection of PJI in tissues obtained from patients with TJA during revision surgery.

**FIG 1:**
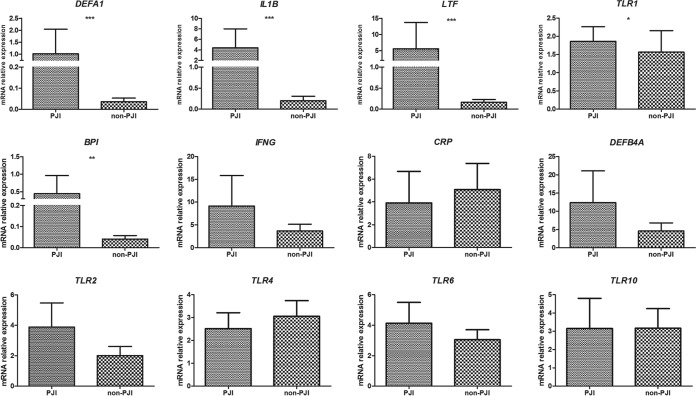
Relative gene expression levels of studied genes in periprosthetic tissues from PJI and non-PJI patients, as determined with the RotorGene Q system. Group means are indicated by horizontal bars, and error bars indicate 95% confidence intervals. Only *P* values for significant differences between groups of patients are stated (*, *P* < 0.05; **, *P* < 0.01; ***, *P* < 0.001).

Among the studied genes, enhanced gene expression of *DEFA1* (*P* < 0.0001), *IL1B* (*P* < 0.0001), *LTF* (*P* < 0.0001), *TLR1* (*P* = 0.02), and *BPI* (*P* = 0.01) was detected for patients with clinically proven PJI compared to that for patients with aseptic loosening (AL) by use of the RotorGene Q system; in addition, high interindividual variability was detected in the patient subgroups (Fig. 1; Table 1). Gene expression levels of *IFNG*, *CRP*, *TLR2*, *TLR4*, *TLR6*, *TLR10*, and *DEFB4A* did not differ between PJI and non-PJI cases (*P* > 0.05) (Fig. 1).

**TABLE 1:** Relative mRNA expression levels of candidate genes in periprosthetic tissues obtained from patients with and without PJI^*a*^

Gene	RotorGene Q			Xxpress		
	Mean (95% CI)		*P* value	Mean (95% CI)		*P* value
	PJI (*n* = 38)	Non-PJI (*n* = 38)		PJI (*n* = 23)	Non-PJI (*n* = 25)	
*DEFA1*	1.021 (0.001–2.042)	0.035 (0.017–0.053)	<0.0001	19.650 (0.278–57.030)	0.057 (0.013–0.102)	<0.0001
*IL1B*	4.361 (0.717–8.006)	0.201 (0.093–0.309)	<0.0001	0.637 (0.042–1.585)	0.013 (−0.0001–0.025)	0.0004
*LTF*	5.599 (0.228–13.710)	0.162 (0.095–0.228)	<0.0001	7.606 (0.638–18.380)	0.397 (0.183–0.612)	0.0003
*TLR1*	1.856 (1.452–2.261)	1.563 (0.970–2.156)	0.019	2.728 (1.313–4.143)	0.927 (0.320–1.535)	0.001
*BPI*	0.445 (0.039–0.964)	0.041 (0.025–0.057)	0.010	1.524 (0.177–3.559)	0.181 (0.020–0.341)	0.002
*IFNG*	9.146 (2.438–15.850)	3.690 (2.230–5.150)	0.644	0.954 (0.405–1.503)	1.681 (0.405–2.958)	0.451
*TLR2*	3.876 (2.276–5.476)	2.005 (1.399–2.610)	0.137	1.680 (0.988–2.373)	0.625 (0.206–1.043)	0.002
*TLR4*	2.515 (1.817–3.213)	3.060 (2.380–3.740)	0.169	7.549 (4.679–10.420)	5.651 (3.316–7.986)	0.358
*CRP*	3.912 (1.151–6.673)	5.079 (2.781–7.377)	0.175	NA	NA	NA
*TLR6*	4.121 (2.748–5.495)	3.045 (2.387–3.703)	0.983	NA	NA	NA
*TLR10*	3.154 (1.504–4.804)	3.164 (2.087–4.242)	0.448	NA	NA	NA
*DEFB4A*	12.350 (3.624–21.080)	4.601 (2.397–6.806)	0.537	NA	NA	NA

a Results are expressed relative to levels of *HPRT1* mRNA. 95% CI, 95% confidence interval; NA, not available.

### Utility of ultrafast qRT-PCR.

In order to assess the utility of ultrafast qRT-PCR for detection of infected tissues within a reasonable time, we introduced gene profiling on the novel qPCR Xxpress instrument. Moreover, gene expression patterns obtained by use of the RotorGene Q and Xxpress instruments were compared.

**FIG 2:**
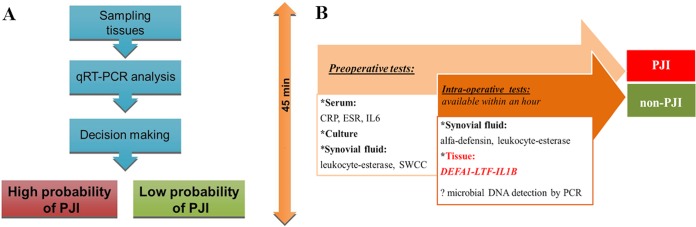
Schema of sample processing from tissue sampling to decision-making, using tissue gene profiling (A) and a diagnostic work-up strategy for exclusion of PJI (B). SWCC, synovial white cell count.

The data from the qRT-PCR Xxpress system were comparable to the data from the RotorGene Q system regarding upregulation/downregulation and significance, but they differed in their obtained threshold cycle (*C_T_*) values (Table 1). A difference was achieved only for *TLR2* gene expression, as the difference between PJI and non-PJI patients reached significance on the Xxpress instrument but not on the RotorGene Q instrument. Using one-step RT-PCR, the qRT-PCR data were available within 20 min. Using the Xxpress instrument, the procedure from sampling to availability of qRT-PCR data takes less than 45 min (Fig. 2A).

### Search for genes and combinations of genes distinguishing PJI from non-PJI tissues.

In order to find genes or combinations of genes that characterize the group of patients with clinically proven PJI, we applied a neural network-based feature selection algorithm. Moreover, we calculated receiver operating characteristic (ROC) curves for *DEFA1*, *IL1B*, *LTF*, and *TLR1* and neural network classification results based on the selected combination of *DEFA1-IL1B-LTF* and the top five neural networks.

**FIG 3:**
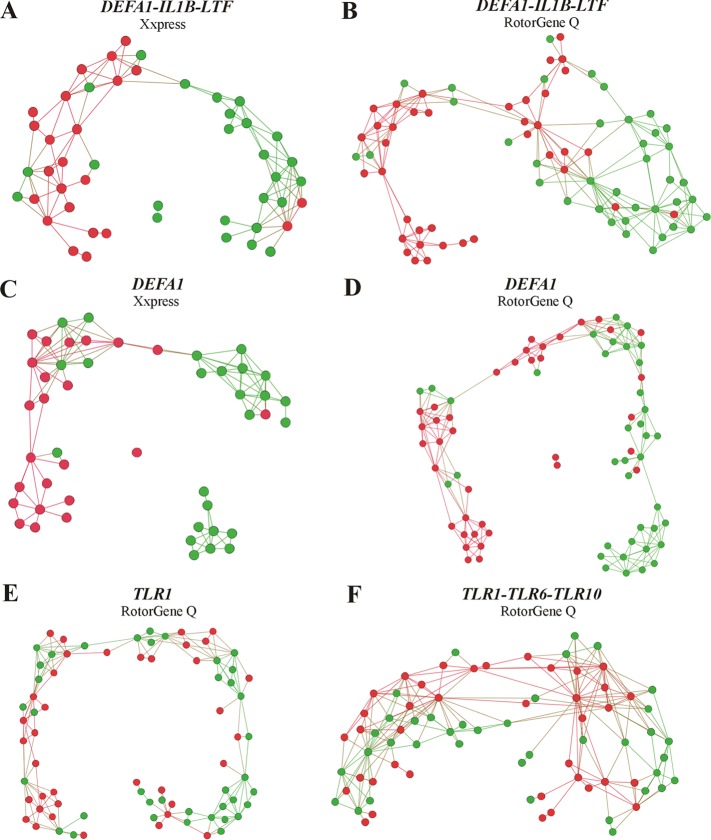
Networks visualizing the best combinations of genes distinguishing PJI from non-PJI cases, using a neural network-based feature selection algorithm. The results are shown for gene patterns of *DEFA1-IL1B-LTF* (A and B), *DEFA1* (C and D), *TLR1* (E), and *TLR1-TLR6-TLR10* (F) obtained with Xxpress (A and C) and RotorGene Q (B and D to F) instruments. Network vertices represent individual gene patterns (individual patient records); edges (links) between the vertices represent similarities of the corresponding patient records, calculated by the Gaussian function. Colors distinguish the classes as patients with clinically proven PJI (red) and non-PJI patients (green).

**FIG 4:**
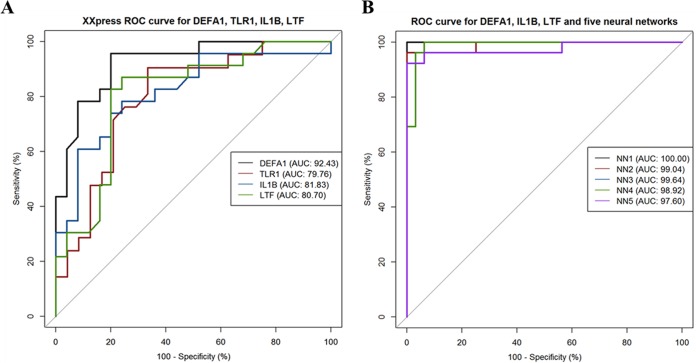
ROC curves with area under the concentration-time curve (AUC) values for *DEFA1*, *IL1B*, *LTF*, and *TLR1* gene profiles (A) and for the top five neural networks for the *DEFA1-IL1B-LTF* combination (B).

The neural network-based feature selection algorithm identified *DEFA1-IL1B-LTF* as the best combination for characterization of clinically proven PJI. Using this gene combination, we were able to achieve >81% overall agreement for all enrolled patients, on average, based on 100 10-fold cross-validated neural networks (Fig. 3). Importantly, all patients with positive microbiology findings were correctly classified into the PJI group (100% positive agreement). For the combination of *DEFA1-IL1B-LTF*, the observed sensitivity and specificity for the top five neural networks reached 94.5% and 95.7%, respectively. The likelihood ratios (LRs) for positive and negative results for the top five neural networks, on average, were 16.3 (95% confidence interval, 15.9 to 16.7) and 0.06 (95% confidence interval, 0.06 to 0.06), respectively (Table 2; Fig. 4).

**TABLE 2:** Value of gene expression profiles of periprosthetic tissues obtained with the Xxpress system for diagnosis of PJI, considering the results for each sample independently^*a*^

Gene(s) tested	Mean % (95% CI)						
	Sensitivity/positive percent agreement	Specificity/negative percent agreement	Positive predictive value	Negative predictive value	Accuracy	LR+	LR−
*DEFA1*	95.7 (95.4–96.0)	79.8 (79.3–80.3)	81.2 (80.8–81.7)	95.3 (95.0–95.6)	87.4 (87.1–87.7)	5.9 (5.6–6.1)	0.05 (0.05–0.06)
*IL1B*	78.1 (77.6–78.7)	75.8 (75.3–76.4)	74.7 (74.1–75.2)	79.2 (78.7–79.7)	76.9 (76.6–77.3)	3.8 (3.7–3.9)	0.29 (0.28–0.30)
*LTF*	82.8 (82.3–83.3)	79.9 (79.4–80.4)	79.3 (78.8–79.8)	83.4 (82.9–83.9)	81.3 (81.0–81.6)	5.0 (4.8–5.2)	0.22 (0.21–0.22)
*TLR1*	90 (89.6–90.4)	66.6 (66.0–67.2)	70.3 (69.7–70.8)	88.3 (87.9–88.8)	77.5 (77.1–77.9)	3.0 (2.9–3.1)	0.15 (0.15–0.16)
*DEFA1-IL1B-LTF* avg for NN1 to NN5	94.5 (94.4–94.7)	95.7 (95.5–95.9)	95.0 (94.8–95.2)	95.6 (95.5–95.8)	95.2 (95.1–95.3)	16.3 (15.9–16.7)	0.06 (0.06–0.06)

a LR+, likelihood ratio for a positive result; LR−, likelihood ratio for a negative result; NN, neural network.

Even the sole expression of *DEFA1* was able to characterize both the PJI and non-PJI groups of patients, with a sensitivity of 95.7% and a specificity of 79.3% (threshold = 0.046) (Table 2; Fig. 3). A neural network with 10-fold cross-validation showed the high utility of *DEFA1* for separation of PJI cases, achieving 80% overall agreement, on average, on both instruments (Fig. 3). For *IL1B* (threshold = 0.005), the sensitivity was 78.1% and the specificity 75.8%; for *LTF* (threshold = 0.483), these values were 82.8% and 79.9%, respectively (Table 2; Fig. 3).

Classification based on neural network analysis also showed that expression of *TLR1* (Fig. 3) or the *TLR1-TLR6-TLR10* combination (Fig. 3) is not usable for classification of PJI and AL. The overall percent agreement reached 49% for *TLR1* and 57% for the *TLR1-TLR6-TLR10* combination; other *TLR* genes (*TLR2*, *TLR4*, *TLR6*, and *TLR10*) and even their combinations did not reach overall agreement levels of >40%. Detection of gene expression of *TLR1* (threshold = 0.616) showed a sensitivity of 90.0% and a specificity of 66.6% (Table 2; Fig. 3).

### Calculator of PJI probability.

**FIG 5:**
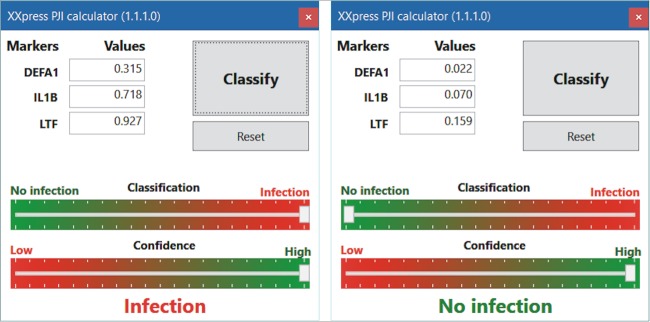
Representative examples of end-user interface results of the electronic calculator for the *DEFA1-IL1B-LTF* gene combination expression data for patients from the test cohort, showing a high probability of PJI (left) and a low probability of PJI (right).

**FIG 6:**
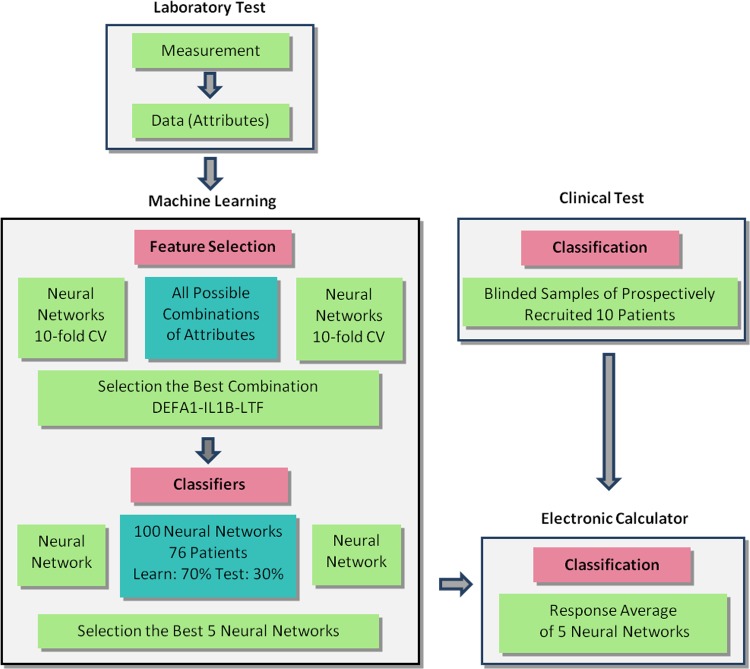
Algorithm flow chart (laboratory tests, neural networks/feature selection algorithm, and clinical testing) used in this study.

To identify patients with PJI, we created an electronic decision tool based on the top five neural networks (Fig. 5). To test the ability of the trained neural network models to identify PJI, we analyzed additional blinded samples from clinical settings that were not previously used for the training procedure (Fig. 6). For the test group of 10 patients, we classified 3 PJI and 7 non-PJI patients correctly (Table 3). Using this tool, we were able to deliver the data to clinicians for intraoperative decision-making immediately after obtaining qRT-PCR results (Fig. 2B).

**TABLE 3:** Characteristics of a “test group” of patients undergoing revision surgery after TJA^*a*^

Patient ID	Patient characteristic		Preoperative test result						Intraoperative test result		MSIS classification
	Gender	Primary diagnosis	CRP concn in serum (mg/liter)	IL-6 concn in serum (pg/ml)	SWCC (10^3^ cells/μl)	Neutrophils in fluid (%)	Lymphocytes in fluid (%)	CRP concn in fluid (mg/liter)	*DEFA1-IL1B-LTF* profiling classification	Confidence (%)	
P1	Male	RA	51.8	10.4	75.5	97.5	2.5	20.3	Infection	90	PJI
P2	Male	POA	31.7	16.8	34.5	92.0	8.0	NA	Infection	60	PJI
P3	Male	POA	29.2	11.2	NA	NA	NA	NA	Infection	100	PJI
P4	Female	PD	7.1	3.3	0.5	31.1	68.9	2.0	No infection	100	Non-PJI
P5	Female	POA	0.6	2.2	NA	NA	NA	NA	No infection	95	Non-PJI
P6	Female	PD	0.3	NA	0.4	54.5	45.5	0.2	No infection	100	Non-PJI
P7	Male	NA	16.1	3.2	0.5	53.6	46.4	1.5	No infection	95	Non-PJI
P8	Female	POA	2.3	7.4	0.3	53.0	47.0	0.7	No infection	100	Non-PJI
P9	Female	POA	3.7	5.6	1.6	27.1	72.9	1.0	No infection	85	Non-PJI
P10	Female	POA	6.3	NA	0.5	50.0	42.0	NA	No infection	63	Non-PJI

a POA, primary osteoarthritis; PD, postdysplastic hip arthritis; RA, inflammatory joint disease, rheumatoid arthritis; SWCC, synovial white cell count; NA, not available.

## DISCUSSION

In this study, we introduced ultrafast qRT-PCR to detect/exclude PJI in patients with TJA. The expression of the *DEFA1-IL1B-LTF* gene combination in periprosthetic tissues changes the pretest probability of PJI markedly within 45 min after sampling, making this examination appropriate for intraoperative decision-making. In addition, we developed a decision support tool for the interpretation of data obtained from expression profiling.

The timely diagnosis of PJI is crucial for intraoperative decision-making, as treatment of PJI necessitates unique surgical strategies compared to those for aseptic reoperation, aiming primarily at eradication of the infecting organisms (15, 16). The majority of infections are easily diagnosed preoperatively by use of more or less traditional diagnostic tools, and this is followed by appropriate therapy (4). However, for patients with low-grade PJI or those that appear to have AL, the probability of PJI can unexpectedly increase intraoperatively, so we need a sensitive and fast diagnostic tool for the rapid and reliable differentiation of true PJI from false-positive results. For this study, we wondered whether expression profiling of genes previously associated with infection may help identify PJI in periprosthetic tissue, especially when synovial fluid is not available but tissues are available. For feature selection and classification of gene expression data in our study, we used a neural network approach shown to have excellent properties for the analysis of gene expression patterns (17–20). These microarray studies proved that a neural network takes into account the intrinsic characteristics of gene expression data, confirms the most informative gene subsets, and improves the classification accuracy with the best parameters based on data sets. Our analysis of the expression profiles of candidate genes previously associated with PJI revealed a high utility of the expression of *DEFA1*, a gene coding for alpha-defensin, for distinguishing patients with and without PJI. This observation is in line with the evidence for an alpha-defensin test of synovial fluid as a highly sensitive and specific PJI biomarker. The alpha-defensin test is in excellent accordance with the Musculoskeletal Infection Society (MSIS) criteria compared to other tests routinely used for the pre-/intraoperative diagnosis of PJI (culture, erythrocyte sedimentation rate [ESR], CRP level, synovial white cell count, and leukocyte-esterase test) (21, 22). This antimicrobial peptide is released mainly by local neutrophils in response to a wide spectrum of microorganisms, regardless of the organism type, Gram type, species, or virulence of the organism (23). Importantly, some studies have proven that alpha-defensin testing maintains its performance for PJI even in the setting of antibiotic administration (22).

Moreover, there is evidence that alpha-defensin testing performs considerably better when it is combined with the synovial CRP level (21). Additionally, our results show that the combination of *DEFA1* with other genes, such as *IL1B*, distinguishes PJI better than *DEFA1* alone does. IL1B is a critical mediator of the host's response to microbial infections and bacterial clearance (24). Its protein levels in synovial fluid were also reported to be highly informative for PJI in patients with systemic inflammatory diseases and preoperative antibiotic treatments (12). Furthermore, LTF possesses antimicrobial activity and is an important part of the innate defense against bacteria, fungi, and viruses (25). There is already evidence that LTF, together with other biomarkers (alpha-defensin, ELA2, BPI, and NGAL), correctly predicts the occurrence of PJI in synovial fluid (26). In our study, the combination of *DEFA1* with *CRP* did not enhance the differentiation between patients with PJI and non-PJI patients, probably as a result of the known nonspecific expression of *CRP* in other cells (e.g., infiltrating immune cells) and/or its involvement mainly in the early defense against infections (27).

Importantly, the gene expression levels of *DEFA1*, *IL1B*, and *LTF* were elevated in all the samples with positive cultures; however, two patients with negative cultures but classified as having PJI were put into the non-PJI group. We suggest that although the tissues from these patients fulfilled the PJI criteria, they may have already been in the late stage of the anti-infective response. Our observation is supported by the fact that the main producers of *DEFA1* and other antimicrobial molecules are short-lived neutrophils, the first immune cells to travel to the sites of bacterial, fungal, and viral infections (28). One may therefore suggest that the tissue gene pattern might already reflect a (different) late stage of response to PJI, while the synovial fluid may still contain elevated levels of soluble PJI biomarkers. On the other hand, five (14%) of the samples without PJI were classified into the PJI group. The enhanced expression of *DEFA1-IL1B-LTF* may deliver a different type of information, reflecting an earlier phase of PJI or, particularly, a low grade of PJI, which may not be detected yet by methods analyzing synovial fluid or serum. The clinical relevance of these observations and their explanation should be elucidated further.

Next, we analyzed the expression patterns of members of the TLR family and other genes known to play a crucial role in the innate immune response to invading pathogens (29). Despite *TLR1* being nominated as a potential marker for PJI in periprosthetic tissues (11), we did not prove its utility for distinguishing PJI from non-PJI cases. Additionally, *TLR2* and other TLR members nominated by previous studies (11, 13) that were examined here did not show any predictive value for the detection of PJI in our patients. The reason may be the involvement of TLRs not only in pathogen recognition but also in wear particle-induced inflammation and osteolysis (30), which may well limit their usage as biomarkers of PJI. Despite their antimicrobial and/or antiviral activities, *BPI*, *DEFB4A*, and *IFNG* did not show utility for the identification of PJI in tissues either.

We believe that gene profiling of periprosthetic tissue might help determine the true stage of tissue damage, including that induced by pathogen invasion. Although our exploratory study needs further testing on a larger cohort of patients, we suggest that tissue expression profiling of a particular set of genes coding for anti-infective proteins may also be useful for the detection of infection in other tissues. Moreover, our data also show utility for distinguishing between tissues with acute infection and those in late resolving and recovery phases. Whether gene profiling of a tissue is suitable for distinguishing between present and past infections in tissues and, particularly, its clinical relevance merit further investigation. This might open up a new avenue for intraoperative diagnostics, including cases before the reimplantation of an implant in two-stage surgery.

This study has several limitations. We are aware that the infection in our patient cohort was based on a definition of PJI that did not involve the parameters of synovial fluid (31). We used a relatively simple histological definition of PJI (32), as the new histological definition (33) was applied for routine specimen analysis later. Additionally, for some patients we did not analyze synovial joint fluid because the joint was dry or synovial joint fluid leaked because of a surgical mistake. Moreover, the current study did not reflect the clinical and causative heterogeneity of PJI (type of bacteria, acuteness versus chronicity of presentation, and duration of symptoms). Thus, this exploratory study showing excellent diagnostic characteristics for the detection of PJI needs to be validated with larger study cohorts diagnosed according to the MSIS criteria and with subgroups reflecting the heterogeneity of PJI.

Taken together, our data show that gene profiling of periprosthetic tissues provides a precise and fast enough method compared to the current detection of PJI biomarkers in synovial fluid and serum. This may be extremely useful in cases where joint fluid is not available or is compromised by blood, etc., and in resolving situations with conflicting data. Our ultrafast approach shows considerable potential as an alternative intraoperative test for surgeons that is intended for the precise and timely diagnosis of PJI.

## MATERIALS AND METHODS

### Study cohort.

The study cohort consisted of Czech Caucasian patients undergoing revision surgery after TJA (*n* = 76) between 2010 and 2016 at a single institution (Department of Orthopaedics, University Hospital, Olomouc, Czech Republic); all patients were operated on by a single surgeon. Among the patients in the cohort, 38 underwent reoperation due to AL and 38 due to PJI. The PJI cases were classified according to the following definition of PJI (31): (i) presence of a sinus tract communicating with a joint and/or intra-articular pus; (ii) coincidentally positive results of histological examination (five or more neutrophils per high-power field [HPF]) and culture of intraoperative samples (≥2 cultures positive for the same microorganism); and (iii) if only intraoperative culture or histological results were positive, then at least two of the following signs had to be present: high clinical suspicion of infection (acute onset, fever, erythema, edema, persistent local pain, early prosthetic failure, wound healing disturbances, etc.), erythrocyte sedimentation rate of >30 mm/h, C-reactive protein level elevated more than 1.5 times above the laboratory reference value, and positive technetium-99m leukocyte scintigraphy. The infections in patients recruited for the test group (*n* = 10) were based on clinical features matching the Musculoskeletal Infection Society (MSIS) criteria (34). The enrolled AL patients did not fulfill the PJI criteria.

Periprosthetic tissue specimens were harvested according to the in-house clinical protocol for histopathological, culture, and immunological examinations. At least 3 samples (range, 3 to 7) for each method under study were taken from each patient, using a surgical knife and forceps. Sampling sites were as follows: (i) inner membrane of a TJA pseudocapsule; (ii) membrane from the bone-implant interface surrounding the proximal component, if available; and (iii) membrane from the bone-implant interface surrounding the distal component, if available. Additionally, one or more samples of tissues with a high suspicion of infection/inflammation were taken from some patients, depending on the surgeon's decision. Tissues from the PJI patients were routinely assessed by culture-based analysis of multiple specimens as described previously (31). For detailed patient characteristics and culture results, see Table 4.

**TABLE 4:** Patient clinical characteristics^*a*^

Characteristic	Value			
	Patients with TJA (*n* = 76)		Patients with TJA, test-blinded data set (*n* = 10)	
	PJI	Non-PJI	PJI	Non-PJI
No. of patients	38	38	3	7
No. of males/females	20/18	9/29	3/0	1/6
No. of patients with TKA/THA	27/11	23/15	2/1	2/5
Interval between index surgery and revision [mo (range)]	40 (0.4–231)	142 (12–303)	17 (2–40)	159 (48–278)
Patient age at index surgery [yr (range)]	65 (40–83)	57 (26–78)	63 (60–70)	57 (43–67)
Patient age at revision [yr (range)]	69 (43–84)	69 (31–89)	65 (60–73)	71 (55–83)
No. of patients with primary diagnosis of:				
Primary osteoarthritis	31	27	2	5
Posttraumatic arthritis	2	2	0	0
Postdysplastic arthritis	2	9	0	2
Avascular necrosis	2	0	0	0
Inflammatory disease	1	0	1	0
No. of patients with indicated type of infection				
Based on time course				
Early postoperative	7		1	
Delayed infection	5		1	
Chronic infection	13		1	
Based on clinical manifestation				
Evident PJI	31		3	
Low-grade PJI	7		0	
Based on pathogenesis				
Surgery related	27		2	
Hematogenous	5		1	
Infection from surrounding environment	5		0	
Recurrent infection	1		0	
No. of patients who were culture positive/negative/NA	25/13/0	0/38/0	3/0/0	0/6/1
No. of patients with positive culture for infectious agent				
Staphylococcus aureus	10		1	
Coagulase-negative Staphylococcus	4		2	
Streptococcus sp.	6		0	
Pseudomonas aeruginosa	3		0	
Mycobacterium tuberculosis	1		0	
Klebsiella pneumoniae	1		0	
No. of patients with histology results (yes/no)	29/9	38/0	3/0	7/0
Noninfectious	11	30	1	6
Infectious (>5 neutrophils per HPF)	18	2	2	0
Undetermined	0	6	0	1
SWCC (10^3^ cells/μl)	54.6 (0.1–267.9)^*f*^	1.4 (0.2–10.1)^*d*^	55.0 (34.5–75.5)^*b*^	0.6 (0.3–1.6)^*b*^
Neutrophils in synovial fluid [% (95% CI)]	72.5 (0.6–98.0)^*g*^	43.7 (18.0–85.0)^*h*^	94.8 (92.0–97.5)^*b*^	44.9 (27.1–54.5)^*b*^
Lymphocytes in synovial fluid [% (95% CI)]	19.0 (0.3–96.0)^*g*^	42.2 (0.06–88.0)^*h*^	5.3 (2.5–8.0)^*b*^	53.8 (42.0–72.9)^*b*^
CRP concn in serum [mg/liter (95% CI)]	105.0 (4.0–371.7)^*e*^	4.5 (0.1–17.0)^*g*^	37.6 (29.2–51.8)^*b*^	5.2 (0.3–16.1)
IL-6 concn in serum [pg/ml (95% CI)]	353.8 (12.6–3,466.0)^*g*^	22.3 (10.6–43.2)^*i*^	12.8 (29.2–51.8)^*b*^	4.3 (2.2–7.4)^*c*^

a The PJI cases were classified as described previously (31); patients enrolled as the “test group” were classified according to the MSIS criteria (34). The types of PJI based on the time course were defined as reported previously (41). Based on clinical evidence, the patients were classified into evident PJI cases, with clinically evident sepsis in terms of clinical manifestation and laboratory tests, and those who had rather hidden PJI (called low-grade PJI), usually with a late onset, less specific symptoms, and lower levels of serum biomarkers. The origin of infection (surgical contamination, hematogenous infection, infection from the surrounding environment, or recurrent infection) was classified as described previously (42). Index surgery, the surgery predating reoperation; TKA, total knee arthroplasty; THA, total hip arthroplasty; SWCC, synovial white cell count; NA, not applicable/not available; HPF, high-power field (magnification, ×400).

b Data were missing for 1 patient.

c Data were missing for 2 patients.

d Data were missing for 4 patients.

e Data were missing for 7 patients.

f Data were missing for 10 patients.

g Data were missing for 13 patients.

h Data were missing for 15 patients.

i Data were missing for 25 patients.

Written informed consent about the usage of periprosthetic tissues for the purpose of this study was obtained from each subject, and the local ethics committee approved the study.

### One-step RT-PCR.

The pseudosynovial tissue samples (0.1 to 1 mg) obtained during revision surgery were placed immediately in Tri reagent (Sigma-Aldrich, Germany) and used immediately for isolation of RNA by use of a Direct-zol RNA miniprep kit (Zymo Research, USA) according to the manufacturer's recommendations. One-step qRT-PCR was performed in a total volume of 20 μl containing Kapa SYBR Fast One-Step qRT-PCR mix (Kapa Biosystems, USA), 200 nM (each) gene-specific primers, and 5 μl of RNA (10 ng/μl; absolute amount, 50 ng). See Table 5 for primer sequences (Integrated DNA Technologies, USA). qRT-PCR was performed on two systems, i.e., the RotorGene Q (Qiagen, USA) and Xxpress (BJS Biotechnologies, United Kingdom) systems. Each run included a no-template control, in which RNA was replaced by water. In detail, for reverse transcription, the sample was held at 42°C for 5 min, followed by enzyme inactivation at 95°C for 3 min. PCR was then performed for 35 cycles of 95°C for 3 s and 60°C for 20 s. qRT-PCR takes 60 min on the RotorGene Q system and 20 min on the Xxpress PCR system. The data were normalized to the housekeeping gene *HPRT1* (35, 36). A human universal reference RNA (Stratagene, USA) was used in triplicate as a calibrator, at 12.5 ng/reaction mix, calculated based on input RNA.

**TABLE 5:** Investigated genes and primers used for qRT-PCR

Gene symbol	Gene product	RefSeq accession no.	Forward/reverse primer sequence (5′ to 3′)	Amplicon length (bp)
*HPRT1*	Hypoxanthine phosphoribosyltransferase 1	NM_000194.2	TGATAGATCCATTCCTATGACTGTAGA/CAAGACATTCTTTCCAGTTAAAGTTG	127
*DEFA1*	Defensin, alpha 1	NM_004084.3	CCTGCCTAGCTAGAGGATCTGT/CATCAGCTCTTGCCTGGAGT	114
*DEFB4A*	Defensin, beta 4A	NM_004942.3	GAGGGAGCCCTTTTCTGAATC/GTCTCCCTGGAACAAAATGC	89
*LTF*	Lactotransferrin	NM_002343.5	CTAATCTGAAAAAGTGCTCAACCTC/GCCATCTTCTTCGGTTTTACTTC	78
*BPI*	Bactericidal/permeability-increasing protein	NM_001725.2	ACGTGCACATCTCAAAGAGC/CGAAGCGCAGACTCAATTTT	73
*IL1B*	Interleukin 1 beta	NM_000576.2	CTAAACAGATGAAGTGCTCC/GGTCATTCTCCTGGAAGG	183
*IFNG*	Gamma interferon	NM_000619.2	GGCATTTTGAAGAATTGGAAAG/TTTGGATGCTCTGGTCATCTT	112
*CRP*	C-reactive protein, pentraxin related	NM_000567.2	GAATTCAGGCCCTTGTATCACT/ACACAAAAGCCTTCCTCGAC	124
*TLR1*	Toll-like receptor 1	NM_003263.3	CCCTACAAAAGGAATCTGTATC/TGCTAGTCATTTTGGAACAC	89
*TLR2*	Toll-like receptor 2	NM_003264.4	CTTTCAACTGGTAGTTGTGG/GGAATGGAGTTTAAAGATCCTG	176
*TLR4*	Toll-like receptor 4	NM_138557.2	GATTTATCCAGGTGTGAAATCC/TATTAAGGTAGAGAGGTGGC	75
*TLR6*	Toll-like receptor 6	NM_006068.4	CTGCCCAAGATTCAGGAGTG/CCATTGCCTTACAACAAAGTTCT	63
*TLR10*	Toll-like receptor 10	NM_030956.3	AGATTGCTTTTGCCACCAAC/TCTCACATCTCCTTTTGATAGCC	114

### Statistical analysis and feature selection algorithm.

Statistical analysis (nonparametric Mann-Whitney U test) of relative gene expression values was performed using GraphPad Prism 5.01 (GraphPad Software, La Jolla, CA, USA). ROC curves, sensitivity, specificity, positive predictive value, negative predictive value, accuracy, and positive/negative likelihood ratios (LRs) for gene expression profiles were calculated using R software. The threshold averaging method on bootstrapped values was used for calculation of confidence intervals (37).

For identification of the best combination of genes distinguishing PJI from non-PJI cases, a neural network-based feature selection algorithm containing the correct class assignment (PJI/non-PJI) for each record of expression values was used. Neural networks are based on features analogous to those of human learning. Depending on the learning (training) data, neural networks have the ability to generalize. This is manifested by the fact that the trained network is able to deduce phenomena that can be derived in some way, even though they were not (or only to a limited extent) part of learning (38).

For visualization of the data set, a transformation of data to the network (graph) was applied. Network vertices represent individual patient records (vectors of gene expression values); edges (links) between the vertices represent the similarities of the corresponding records based on the Gaussian function. The edges were chosen to link the nearest neighbors (vertices having the highest similarities). The number of the nearest neighbors for each vertex corresponds to vertex representativeness (39).

### Classification of patients by the artificial neural network.

The artificial neural network (Neuralnet package [https://cran.r-project.org/package=neuralnet] from R software) was used to find combinations of attributes (i.e., genes) having a high ability to classify patients into two classes (PJI and non-PJI). For all possible combinations of attributes and their 10-fold cross-validations (40), neural networks were trained by using learning data on two hidden layers (13 and 9 neurons), with a backpropagation algorithm and a logistic activation function. For learning and testing, 90% (68 of 76 patients) and 10% (8 of 76 patients) of patients were used for each network at each step of the cross-validation. Only neural networks with a classification error of <20% were then selected. From the corresponding combinations, three top-ranked (based on their *P* values) and most-often-occurring genes, *DEFA1*, *IL1B*, and *LTF*, were selected for classification.

In the next step, 76 patients were used, and 100 neural networks were trained for the selected *DEFA1-IL1B-LTF* gene combination. For the learning of each of 100 networks, 70% of randomly selected patients (53 of 76 patients) were chosen, and the remaining 30% (23 of 76 patients) were used for testing. Because of the random initialization of the network before the start of learning and the different learning and testing sets, each network provided slightly different results for classification. From the total of 100 networks, the five with the smallest classification errors (with the lowest mean square error values) were selected for further use in the electronic calculator. A flow chart of the process is documented in Fig. 6.

### Infection calculator application.

The infection calculator enables one to insert *DEFA1*, *IL1B*, and *LTF* expression values into a small MS Windows application to show the patient's probability of PJI. The classification algorithm is included in the R script and uses the Neuralnet package and the top five fully trained neural networks. Results from the calculator are presented to the end user by two indicators (green-red) for (i) patient classification (infection/no infection) and (ii) the confidence interval, based on values resulting from the obtained classification.
